# Remote Management of Patients With Heart Failure in Medically Underserved Areas

**DOI:** 10.1016/j.jacadv.2026.102676

**Published:** 2026-03-19

**Authors:** Jeremy Florence, Sylvain Ploux, Clément Riocreux, F. Daniel Ramirez, Solenn Toupin, Théo Pezel, Guillaume Clerfond, Romain Eschalier

**Affiliations:** a Cardiology Department, CHU Clermont-Ferrand, Clermont-Ferrand, France; b MIRACL.ai laboratory, Multimodality Imaging for Research and Analysis Core Laboratory and Artificial Intelligence, University Hospital of Lariboisière (AP-HP), Paris, France; c Bordeaux University Hospital (CHU), Cardio-Thoracic Unit, Pessac, France; d IHU Liryc, Electrophysiology and Heart Modeling Institute, Université de Bordeaux, Bordeaux, France; e Division of Cardiology, University of Ottawa Heart Institute, Ottawa, Ontario, Canada; f School of Epidemiology and Public Health, University of Ottawa, Ottawa, Ontario, Canada; g Université Paris Cité (UPC), Department of Cardiology, University Hospital of Lariboisière, (Assistance Publique des Hôpitaux de Paris, AP-HP), Inserm MASCOT-UMRS 942, Paris, France; h Université Clermont Auvergne, CHU Clermont-Ferrand, CNRS, SIGMA Clermont, Institut Pascal, Clermont-Ferrand, France

**Keywords:** heart failure, hospitalization, medically underserved area, remote monitoring, telemonitoring

## Abstract

**Background:**

Medically underserved areas (MUAs) are associated with higher rates of hospitalizations and mortality. Although structured remote management (RM) programs demonstrated clinical benefits, their effectiveness in MUAs in Europe remains scarcely investigated.

**Objectives:**

The objective of the study was to assess whether heart failure (HF) patients derive similar outcomes from a structured, multiparametric, RM program irrespective of MUA designation.

**Methods:**

Consecutive patients enrolled in a standardized, multiparametric, HF RM program between April 2020 and December 2022 in 2 French regions (Auvergne Rhône-Alpes and Nouvelle-Aquitaine) at 2 French university hospitals were included in the study. Inclusion criteria were chronic HF with ≥1 episode of HF hospitalization within the last year or NYHA functional class ≥II associated with an elevated B-type natriuretic peptide. Patient assessments were performed remotely with body weight, blood pressure, heart rate, symptoms, biology, and data from cardiac implantable electronic devices. The primary outcome was the composite of unplanned HF hospitalization or all-cause mortality.

**Results:**

Among 1,040 patients (mean age 72 ± 12 years, 70% male), 32% lived in MUAs. The median follow-up was 20 (IQR: 10-24) months. The annualized rate of the primary outcome was 13.7% (95% CI: 11.9-15.9) in the overall population, with no significant difference between MUAs and no MUAs patients (13.5% [95% CI: 10.3-17.4] vs 13.9% [95% CI: 11.6-16.5]; *P* = 0.876). MUAs were not associated with the primary outcome (adjusted HR: 0.93; 95% CI: 0.68-1.27; *P* = 0.84). Using Kaplan-Meier analysis, survival curves showed no difference between MUA and non-MUA patients (*P* = 0.83).

**Conclusions:**

Our study showed no difference in primary outcome among HF patients enrolled in a structured, multiparametric RM program, irrespective of MUAs.

Individuals living in a medically underserved area (MUA) are at a greater risk of having unmet health care needs due to a scarcity of local health care providers and limited access to medical facilities for specialty care.^1^ These barriers to accessing vital medical services have significant impacts on health outcomes.^2^^,^^3^ In recent years, and especially since the COVID-19 pandemic, innovations in telehealth have shown promise for expanding treatment capacity in MUAs.^4^ Remote management (RM) of heart failure (HF) using telemedical centers to deliver personalized care remotely is among the most advanced of such telehealth solutions. Data demonstrating that RM could reduce unplanned cardiovascular hospital admissions and all-cause mortality prompted the French government to promote its use among patients with HF.^5^^,^^6^ An expected benefit of its adoption is improving access to care in rural areas and MUAs, which are considered priority targets. However, effective RM of patients with HF is expected to require close collaboration between RM centers and local health care professionals. The question then arises as to whether an RM program is an effective strategy for HF patients living in areas that are, by definition, deprived of such local care support. Indeed, multidisciplinary coordination of care for these patients remains complex and sociogeographic nuances could affect program effectiveness at regional and individual levels.^7^ We therefore sought to assess whether HF patients derive similar outcomes from a structured, multiparametric, RM program irrespective of MUAs designation.

## Methods

### Study population

**Figure 1:**
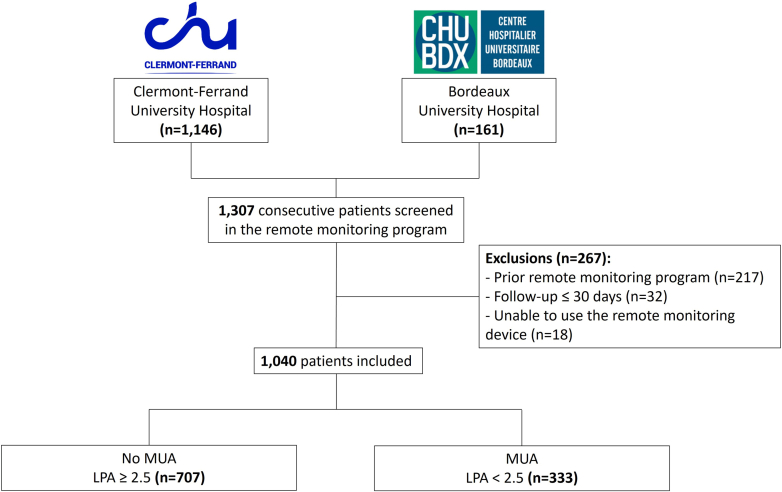
**Study Flowchart** The threshold of LPA <2.5 consultations and/or visits per inhabitant per year was used to define MUA. LPA = localized potential accessibility; MUA = medically underserved area.

Between April 2020 and December 2022, we conducted an observational study of all consecutive patients enrolled in the same multiparametric HF RM program in 2 French regions (Auvergne-Rhône-Alpes and Nouvelle-Aquitaine) at 2 French centers (University Hospital of Clermont-Ferrand and University Hospital of Bordeaux). The inclusion criteria were a diagnosis of chronic HF with at least 1 episode of HF hospitalization within the previous year or NYHA functional class status ≥ II associated with an elevated B-type natriuretic peptide (BNP) >100 pg/mL or N-terminal-pro-BNP (NTproBNP) >1,000 pg/mL. These criteria were consistent with the French government’s eligibility criteria for the national HF RM program. Etiologies of HF were defined by the latest recommendations of the European Society of Cardiology.^6^ Exclusion criteria were: 1) age <18 years; 2) prior enrollment in a different RM program; 3) active pregnancy or breast-feeding, 4) physical or mental factors deemed to render the patient unable to follow the RM program; 5) legal protection measures that precluded patient enrollment; 6) patient refusal to transmit data or to receive relevant teaching, and; 7) follow-up ≤30 days. A detailed study flowchart is shown in Figure 1. The study was approved by the local Ethics Committee (IRB00013412) of our institutions and conducted in accordance with the Declaration of Helsinki. This study followed the STROBE (Strengthening the Reporting of Observational Studies in Epidemiology) reporting guidelines for cohort studies.

### Definition of medically underserved areas

In 2012, the French Public Health Agency developed an index to measure local accessibility for health care, termed “Localized Potential Accessibility” (LPA), which has been widely adopted in France.^8^ Briefly, this index considers the proximity and availability of general practitioners (GPs) as well as an estimate of their workload, combined with health care needs according to the age of the local population. The total number of consultations and/or patient visits for a GP in a given year is divided by the number of inhabitants in their respective area. Each GP can provide multiple consultations or visits to patients, and inhabitants are weighted by age and distance. This yields an estimate of the number of visits to local GPs available per inhabitant. A high LPA index indicates high availability of GPs and favorable access to care in the area, whereas a low LPA index indicates restricted access to GPs and poor access to care. As in previous studies, we defined MUAs as areas with a threshold of <2.5 consultations and/or visits per inhabitant per year.^8^^,^^9^ For each patient in our study, we cross-referenced their primary address of residency with open-source, city-specific LPA values that are publicly available on the French Public Health government website.^10^

### Remote management strategy

RM was performed using the CareLine platform (CareLine Solutions), which collects data on clinical parameters (weight, systolic and diastolic blood pressure measurements, and heart rate), symptom status (using a health status survey), pacemaker/implantable cardioverter-defibrillator (ICD) data, and blood test results (NT-proBNP/BNP, creatinine, urea, potassium, sodium, hemoglobin, and ferritin). Patients were given a scale to monitor their weight, as well as blood pressure and heart rate measuring devices capable of automatic wireless data transmission to a dedicated app (CareLine Solutions). Clinical parameters were collected daily, symptoms were assessed weekly, and blood tests results were evaluated as needed. Transmissions triggered by alerts from pacemakers/ICDs were automatically imported, as were the results of routine remote interrogations. Clinical, biological, and device-related parameters could be incorporated into individual customizable alerts, which were reviewed during office hours (weekdays between 8:00 AM and 6:00 PM) by a specialized team of nurses and physicians at both centers. These alerts, alongside trends in relevant parameters, were used to remotely monitor patients and assess their responses to remote medical interventions, 2 key aspects of their RM. Patient education and lifestyle advice were systematically provided and emphasized during telephone interactions or during e-learning sessions by the dedicated team.

### Patient follow-up and clinical outcomes

The primary outcome was a first event analysis of a composite outcome including unplanned HF hospitalization and all-cause mortality. Secondary outcomes were: 1) “all-event” analysis of all-cause mortality and unplanned HF hospitalization; 2) unplanned HF hospitalization; 3) all-cause mortality; 4) duration of unplanned HF hospitalization; 5) severity of HF when hospitalized; and 6) emergency room admission. Baseline data, including medical history, active treatments, clinical assessments, and symptoms were collected on the day of RM program enrollment. Clinical events were prospectively annotated by the care team within the RM platform. For adjudication purposes, the RM platform records and hospital electronic health records were retrospectively screened by 2 senior cardiologists for HF hospitalizations and all-cause mortality. Interruptions in communication with the platform also prompted investigations for possible HF hospitalizations occurring in other hospitals. Follow-up data were collected up to February 2023.

### Statistical analysis

Continuous variables are expressed as mean ± SD or median (IQR) and compared with Student’s *t*-test or Mann-Whitney *U* test after assessing normality by visual assessment. Categorical variables are presented as n (%) and compared with chi-square or Fisher exact test as appropriate. Adherence was defined as (days with body weight transmission/total follow-up days not spent hospitalized) × 100. Missing data characteristics and patterns were examined, and logistic regression models were used to identify predictors of missingness. Missing at random mechanism was assumed and multiple imputations were performed accordingly with predictors of missingness and outcomes included in the imputation model. The methodology for imputation is presented in Supplemental Tables 1 and 2. Annualized rates were expressed as the ratio of the number of patients who experienced the event divided by the total years of patient follow-up at risk expressed as patient-year. Survival probabilities were estimated using the Kaplan-Meier method and compared with the log-rank test. Cox proportional hazards methods were used to identify the predictors of the primary outcome. Multicollinearity was assessed using variance inflation factors; all values were <1.5, indicating no relevant multicollinearity. The proportional hazard assumption was assessed using Schoenfeld residuals, and the log-linearity was assessed by the Martingale residuals. The primary outcome was also analyzed with Fine and Gray subdistribution hazard ratio (sHR) models to analyze time to first unplanned HF hospitalization, treating all-cause mortality as a competing risk. A complete-case Cox proportional hazards model including only patients without missing data on model covariates was performed as a sensitivity analysis. The secondary outcome analysis was conducted with “all-event” analysis as a comparison of the number of all-cause deaths and unplanned HF hospitalizations divided by the effective duration of follow-up between the 2 groups. Because recurrent HF hospitalizations count exhibited overdispersion, negative binomial regression was used. Deaths that occurred during hospitalization with an overnight stay were counted as 2 events. This approach was intended to capture the total burden of disease-related events.^11^ A subgroup analysis was performed in prespecified subgroups using multiplicative interaction, with *P* values for interaction reported. We used a 2-tailed *P* value <0.05 for statistical significance. Statistical analysis was performed using R software (version R 4.4.0, R Project for Statistical Computing).

## Results

### Study population

Between April 2020 and December 2022, 1,307 patients were enrolled in the RM program at both participating centers. After applying the exclusion criteria, 1,040 patients constituted our study cohort and were analyzed. Overall, 879 patients (85%; 879/1,040) were recruited at University Hospital of Clermont-Ferrand and 161 patients (15%; 161/1,040) at University Hospital of Bordeaux. The baseline characteristics of patients are presented in Table 1. In short, patients were 72 ± 12 years old and 70% (727/1,040) were males. Common comorbidities included atrial fibrillation (68%; 706/1,040), hypertension (67%; 701/1,040), chronic kidney disease (61%; 630/1,040), diabetes (41%; 425/1,040), anemia (29%; 299/1,040), and malnutrition (20%; 210/1,040). The main etiologies for HF were ischemic cardiomyopathy and dilated cardiomyopathy representing 41% (424/1,040) and 21% (218/1,040) of patients, respectively. The distribution of other HF etiologies is represented in Supplemental Figure 1. Inclusion in the RM program occurred following an HF hospitalization for 34% (349/1,040) of patients vs 66% (691/1,040) were outpatient at inclusion. The mean left ventricular ejection fraction (LVEF) was 38%, with 74% (767/1,040) of patients having an LVEF <50%. NYHA functional class status was >II for 39% (407/1,040) of patients and the mean dose of furosemide at baseline was 125 mg daily.

**Table 1:** Baseline Clinical Characteristics of Patients According to MUA in the Overall Population (N = 1,040)

	All Patients (N = 1,040)	No MUA (n = 707)	MUA (n = 333)	*P* Value
Age, n (%)				
Mean ± SD	72 ± 11.9	72 ± 12	72 ± 11.6	0.972
<60 years	158 (15.2)	109 (15.4)	49 (14.7)	
60-80 years	575 (55.3)	388 (54.9)	187 (56.2)	
≥80 years	307 (29.5)	210 (29.7)	97 (29.1)	
Male, n (%)	725 (69.9)	496 (70.2)	231 (69.4)	0.853
NYHA functional class status, n (%)				0.726
I	65 (6.3)	43 (6.1)	22 (6.6)	
II	568 (54.6)	389 (55)	179 (53.8)	
III	381 (36.6)	255 (36.1)	126 (37.8)	
IV	26 (2.5)	20 (2.8)	6 (1.8)	
HF etiologies, n (%)				
Ischemic	424 (40.8)	284 (40.2)	140 (42.2)	0.613
Dilated cardiomyopathy	218 (21)	151 (21.4)	67 (20.1)	0.707
Others	398 (38.2)	272 (38.4)	126 (37.7)	0.825
Comorbidities, n (%)				
Diabetes	425 (40.9)	303 (42.9)	122 (36.6)	0.066
Hypertension	701 (67.4)	483 (68.3)	218 (65.5)	0.398
Chronic kidney disease^a^	630 (60.6)	435 (61.5)	195 (58.6)	0.397
Atrial fibrillation	706 (67.9)	489 (69.2)	217 (65.2)	0.223
Anemia^b^	299 (28.7)	217 (30.7)	82 (24.6)	0.052
Malnutrition^c^	210 (20.1)	158 (22.3)	52 (15.6)	**0.015**
Biology				
eGFR, mL/min/1.73 m^2^	55.2 ± 23.2	54.8 ± 23.6	56 ± 22.5	0.412
NTproBNP, pg/mL	1,867 (838-3,500)	1,966 (858-3,538)	1,712 (789-3,445)	0.260
BNP, pg/mL	320 (193-865)	388 (206-896)	218 (67-271)	0.241
Hemoglobin, g/dL	11.5 ± 2.3	11.5 ± 2.3	11.6 ± 2.2	0.423
Albumin, g/L	43.2 ± 15.1	42.9 ± 15.3	43.9 ± 14.8	0.344
LVEF, mean ± SD	37.7 ± 13.9	37.5 ± 13.4	38.1 ± 14.9	0.548
<50%, n (%)	767 (73.8)	527 (74.5)	240 (72.1)	0.442
HF treatments, n (%)				
ACEi/ARB/sacubitril-valsartan	828 (79.6)	581 (82.2)	247 (74.2)	**0.004**
Beta-blockers	835 (80.3)	567 (80.2)	268 (80.5)	0.981
Aldosterone antagonists	587 (56.4)	395 (55.9)	192 (57.7)	0.634
SGLT2 inhibitors	148 (14.2)	107 (15.1)	41 (12.3)	0.263
Digoxine	12 (1.4)	4 (0.7)	8 (2.5)	**0.036**
Ivabradine	7 (0.8)	5 (0.9)	2 (0.6)	0.988
Furosemide mg, mean ± SD	72 (40-125)	71 (40-125)	72 (20-125)	0.174
Bumetanide	9 (1)	7 (1.2)	2 (0.6)	0.499
Cardiac devices, n (%)				0.718
No cardiac device	422 (40.6)	281 (39.7)	141 (42.3)	
Permanent pacemaker	94 (9.1)	61 (8.7)	33 (10)	
ICD	256 (24.6)	177 (25)	79 (23.7)	
CRT-P	44 (4.2)	33 (4.7)	11 (3.3)	
CRT-D	224 (21.5)	155 (21.9)	69 (20.7)	
Patient status at inclusion, n (%)				0.503
Outpatient	691 (66.4)	475 (67.2)	216 (64.9)	
HF hospitalization	349 (33.6)	232 (32.8)	117 (35.1)	

ACEi = angiotensin-converting enzyme; ARB = angiotensin II receptor; BNP = B-type natriuretic peptide; CRT = cardiac resynchronization therapy; eGFR = estimated glomerular filtration rate; ICD = implantable cardioverter defibrillator; HF = heart failure; LVEF = left ventricular ejection fraction; MUA = medically underserved areas; NT-proBNP = N terminal pro brain natriuretic peptide; SGLT = sodium-glucose cotransporter 2 inhibitors.

Values are n (%), mean ± SD, or median (IQR). **Bold** values indicate the 2-tailed *P* value reached statistical significance (<0.05).

Patients can have mixed HF etiologies.

a Defined by eGFR ≤60 mL/min/1.73 m^2^ twice.

b Defined by hemoglobin <13 g/dL for men and <12 g/dL for women.

c Defined by albumin <30 g/dL.

**Figure 2:**
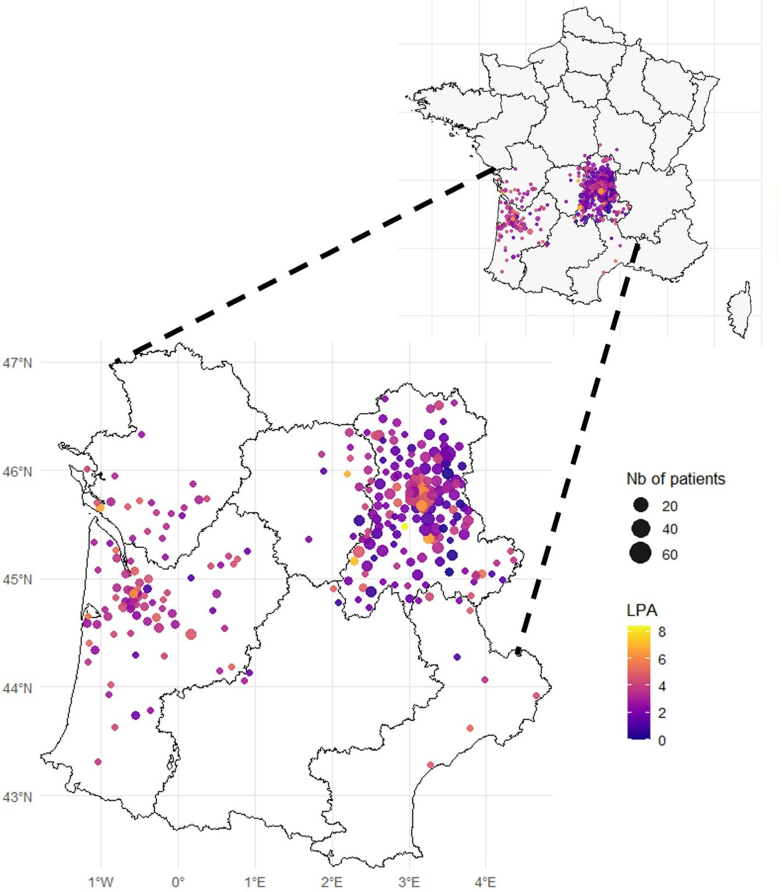
**Localized Potential Accessibility Mapping for Study Patients Across France** Map showing postal code–level geographic accessibility across France and zoom in six southwestern regions. Black lines delineate regional borders. Each circle represents a postal code area, with its size proportional to the number of included patients and its color indicating the LPA. Higher LPA values reflect better access to general practitioners. Abbreviation as in Figure 1.

The distribution of the LPA index in our study population (mean: 3.4; 1st quartile: 2.2; 3rd quartile: 4.6; median: 3.5) was similar to the national distribution LPA (mean; 3.1; 1st quartile: 2.3; 3rd quartile: 3.8; median: 3.1). The distribution map of the LPA index is illustrated in Figure 2. The cohort was dichotomized based on MUA designation. Patients residing in MUAs (n = 333) were overall comparable to patients from non-MUAs (n = 707) (age 72 ± 12 years vs 72 ± 12 years; 69% [231/333] vs 70% [496/707] males). However, patients in MUAs were less likely to suffer from malnutrition (16% [52/333] vs 22% [158/707]; *P* = 0.015) and to receive angiotensin-converting enzyme inhibitors/angiotensin II receptor antagonists/sacubitril-valsartan treatment (74% [247/333] vs 82% [581/707]; *P* = 0.004), but were more likely to receive digoxin (2.5% [8/333] vs 0.7% [4/707]; *P* = 0.036) (Table 1).

### Remote management parameters

RM parameters are shown in Table 2. All patients had daily weight monitoring, patients in MUAs had more blood pressure monitoring (100% [333/333] vs 97% [687/707]; *P* = 0.014), more heart rate monitoring (51% [170/333] vs 36% [256/707]; *P* < 0.001), less symptom monitoring (5% [15/333] vs 17% [123/707]; *P* < 0.001) and less pacemaker/ICD monitoring (3% [10/303] vs 8% [54/707]; *P* = 0.006) than patients residing outside MUAs. Overall adherence to the RM program was 69% (95% CI: 67.1-70.7), with 64% (665/1,040) of patients achieving >70% adherence, with no significant difference based on MUA designation. There was no significant difference in terms of the mean numbers of alerts for all parameters collected during the follow-up between MUA and no MUA patients, except for symptoms (27 vs 21; *P* = 0.006) (Supplemental Table 3).

**Table 2:** Remote Management Parameters Used in the Overall Population (N = 1,040)

	All Patients (N = 1,040)	Non-MUA (n = 707)	MUA (n = 333)	*P* Value
Remote monitoring parameters^a^, n (%)				
Body weight	1,040 (100)	707 (100)	333 (100)	NA
Blood pressure	1,019 (98)	687 (97.2)	332 (99.7)	**0.014**
Heart rate	426 (41)	256 (36.2)	170 (51.1)	**<0.001**
Symptoms	138 (13.3)	123 (17.4)	15 (4.5)	**<0.001**
Cardiac devices	64 (6.2)	54 (7.6)	10 (3)	**0.006**
Remote monitoring adherence^b^, % (95% CI)	69 (67-71)	70 (68-72)	67 (63-70)	0.114

Abbreviation as in Table 1.

Values are n (%) or n (95% CI). **Bold** values indicate the 2-tailed *P* value reached statistical significance (<0.05).

a Remote monitoring parameter corresponds to each parameter measured from the patient and transmitted to the medical team, used to manage patients.

b Remote monitoring adherence was calculated as the percentage of days with transmitted body weight data divided by all effective surveillance days (length of follow-up minus the duration of hospitalizations).

### Prognostic value of residing in MUAs

Over a median follow-up of 20 (10-24) months, the annualized rate of the primary outcome was 13.7% (95% CI: 11.9-15.9) in the overall population, with no significant difference between MUA and no MUA patients (13.5% [95% CI: 10.3-17.4] vs 13.9% [95% CI: 11.6-16.5]; *P* = 0.876).

**Figure 3:**
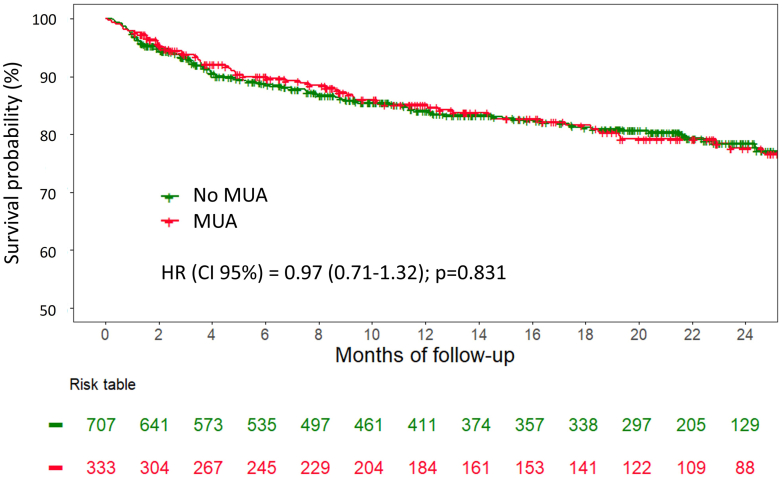
**Survival Curves According to Medically Underserved Area for the Primary Outcome** Kaplan-Meier curves according to MUA for the composite primary outcome (unplanned heart failure hospitalization or all-cause mortality) in the overall population (N = 1,040). Abbreviation as in Figure 1.

**Figure 4:**
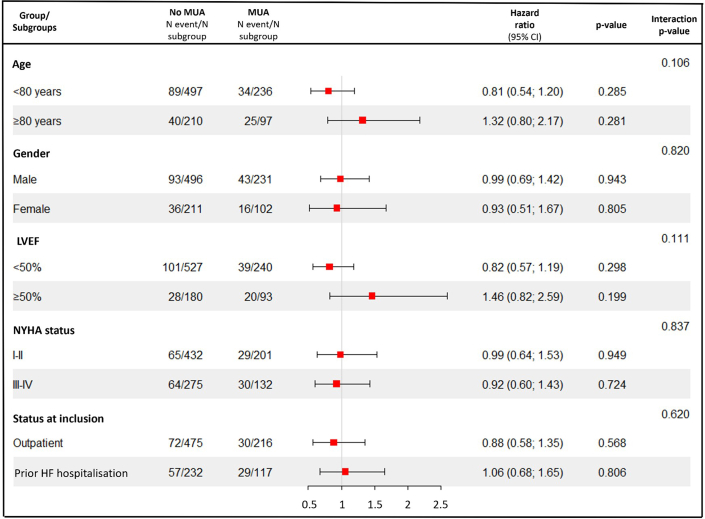
**Subgroups Analysis** Forest plot of incidence of the primary outcome according to MUA in prespecified subgroups. N events/N subgroup: number of patients with the primary outcome/number of patients in the subgroup. LVEF = left ventricular ejection fraction; N: number; HF = heart failure; other abbreviation as in Figure 1.

In univariable analysis, age, NYHA functional class status >II, estimated glomerular filtration rate, anemia, malnutrition, NTproBNP, and baseline dose of furosemide were all significantly associated with the primary outcome, whereas MUA designation was not (HR: 0.97; 95% CI: 0.71-1.32; *P* = 0.829) (Supplemental Table 4). In multivariable analysis with literature-based selection method for adjustment, baseline dose of furosemide, NYHA functional class status >II, estimated glomerular filtration rate, and NTproBNP remained significantly associated with the primary outcome, and MUA designation remained nonsignificant (adjusted HR: 0.93; 95% CI: 0.68-1.27; *P* = 0.839) (Table 3). A complete-case analysis was performed as a sensitivity analysis and yielded consistent results, with no significant change in the association between MUAs and the primary outcome (Supplemental Table 5). Using Kaplan-Meier analysis, survival curves stratified by MUA designation showed no difference in the primary outcome (Figure 3) (log-rank *P* = 0.831). In competing risk analysis, MUAs were not associated with HF hospitalization (Supplemental Figure 2) (sHR: 1.01; 95% CI: 0.88-1.16; *P* = 0.85). These results were consistent across different subgroups of clinical interest, including age, sex, LVEF, NYHA functional class status, and prior HF hospitalization before inclusion in the study (Figure 4).

**Table 3:** Univariable and Multivariable Cox Regression Analyses to Predict the Primary Outcome in the Overall Population (N = 1,040)

	Univariable Analysis		Multivariable Analysis	
	HR (95% CI)	*P* Value	HR (95% CI)	*P* Value
MUA	0.97 (0.71-1.32)	0.829	0.93 (0.68-1.27)	0.839
Age, y	1.02 (1.01-1.03)	**0.002**	0.99 (0.98-1.01)	0.458
Furosemide, per 40 mg increase	1.06 (1.03-1.08)	**<0.001**	1.04 (1.01-1.07)	**0.007**
Other etiologies of HF^a^	0.95 (0.70-1.28)	0.725	0.71 (0.49-1.02)	0.061
Atrial fibrillation	1.17 (0.85-1.60)	0.336	0.89 (0.63-1.25)	0.489
NYHA functional class status >II	1.64 (1.23-2.18)	**0.001**	1.41 (1.03-1.86)	**0.022**
LVEF, per 5% increase	1.05 (0.99-1.11)	0.055	1.01 (0.99-1.02)	0.068
Diabetes	1.17 (0.88-1.56)	0.282	1.17 (0.86-1.59)	0.324
Hypertension	1.10 (0.80-1.49)	**0.561**	0.81 (0.58-1.14)	0.221
eGFR, per 10 mL/min/1.73 m^2^ increase	0.83 (0.77-0.89)	**<0.001**	0.89 (0.83-0.98)	**0.011**
NTproBNP, per 500 pg/mL increase	1.05 (1.04-1.06)	**<0.001**	1.04 (1.03-1.06)	**<0.001**

Abbreviations as in Table 1.

Values are n (%), mean ± SD, **Bold**. values indicate the 2-tailed *P* value reached statistical significance (<0.05).

Selection of covariables for the adjustment was made on a literature-based method.

a Other etiologies of HF correspond to nonischemic nor dilated etiologies of HF.

### Secondary outcome analysis

**Figure 5:**
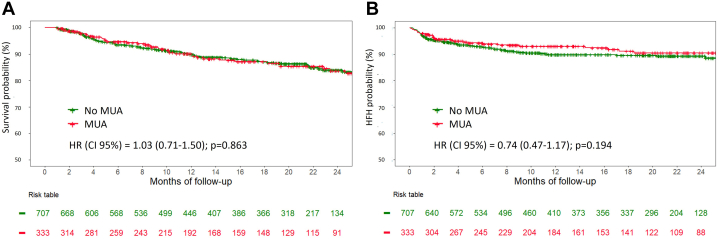
**Survival Curves According to Medically Underserved Area for the Secondary Outcomes** (A) all-cause mortality; (B) Unplanned heart failure hospitalization. Kaplan-Meier curves according to MUA for the secondary outcomes in the overall population (N = 1,040). HFH, heart failure hospitalization; other abbreviation as in Figure 1.

In the overall population, the annualized rates of all-cause mortality and unplanned HF hospitalization were 8.8% (95% CI: 7.3-10.5) and 7.1% (95% CI: 5.7-8.6), respectively. Patients in MUAs had similar annualized rates of all-cause mortality and unplanned HF hospitalization compared to patients living outside MUAs (8.9% [95% CI: 6.4-12.1] vs 8.7% [95% CI: 7.0-10.7], *P* = 0.92; and 5.7% [95% CI: 3.7-8.4] vs 7.7% [95% CI: 6.0-9.7], *P* = 0.23, respectively). In the all-events analysis, patients living in MUAs experienced 0.24 events (including HF hospitalizations and all-cause mortality) per patient-year (95% CI: 0.20-0.29) compared with 0.27 events per patient-year (95% CI: 0.24-0.30) in non-MUA patients (rate ratio: 0.98; 95% CI: 0.72-1.33; *P* = 0.895). Using Kaplan-Meier analysis, survival curves stratified by MUA designation showed no differences in the risk of all-cause mortality or unplanned HF hospitalization (Figures 5A and 5B) (log-rank *P* = 0.863 and *P* = 0.194, respectively). Overall, the median length of stay in hospital for unplanned HF hospitalization was 11 (8-27) days, which did not differ based on MUA designation (*P* = 0.614). There was similarly no difference in the emergency room admission rate between MUA and non-MUA patients (6.3% [21/303] vs 8.2% [58/707]; *P* = 0.341) or in the severity of HF during hospitalizations, with 5 patients (1.5%) vs 9 patients (1.6%) experiencing acute pulmonary edema and 3 patients (1%) vs 9 patients (1.6%) experiencing cardiogenic shock in MUAs vs outside MUAs, respectively (all *P* > 0.05). The result of the study is illustrated in Central Illustration.

**Central Illustration:**
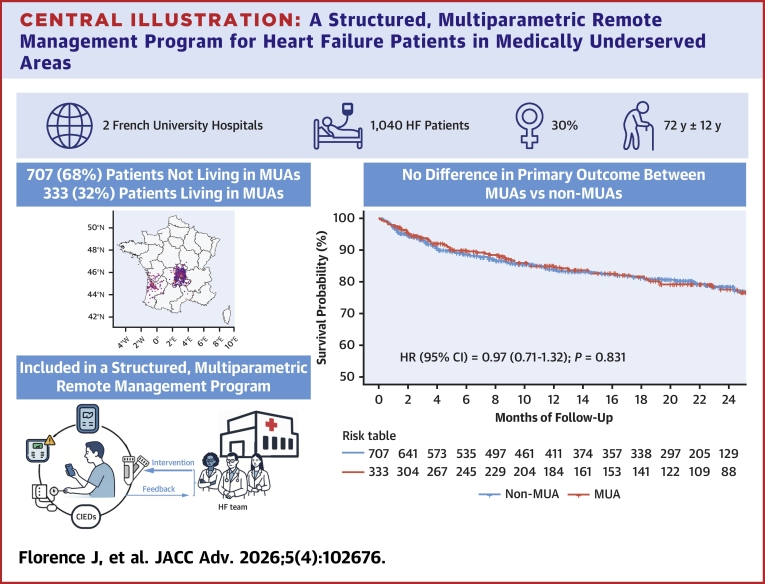
A Structured, Multiparametric Remote Management Program for Heart Failure Patients in Medically Underserved Areas HF = heart failure; MUA = medically underserved area.

## Discussion

In our study of a large population of HF patients enrolled in a multiparametric RM program over a median follow-up of 20 months, the main findings were as follows: 1) the overall annualized rate of the primary outcome was 13.7%; and 2) 32% of patients resided in a MUA, a characteristic that was not significantly associated with the primary outcome.

### Remote management effect on outcomes

HF hospitalizations account for 65% of all HF-related medical costs in Europe and are the primary determinant of impaired quality of life among patients with HF. Reductions in HF hospitalizations are therefore among the most reported outcomes in relevant randomized controlled trials.^12^ In our study, the annualized rate of the primary outcome of 13.7% was markedly lower than previously reported in HF populations not participating in an RM program. Specifically, the annualized rate of unplanned HF hospitalization and all-cause mortality in our cohort were 7.1% and 8.8% in the overall population, respectively. In comparison, recent data from the French national health insurance database reported a 1-year HF readmission rate of 22% and 1-year mortality rates of 14% to 35%.^13^^,^^14^ Additional analyses using nationwide administrative data show even higher 1-year event rates in real-world patients hospitalized for HF, including 31% HF readmissions and 29% mortality.^15^ Real-world French RM cohorts have reported consistent findings: the TELESAT-HF [Evaluating the Effectiveness of Remote Patient Monitoring for Patients With Heart Failure] national analysis observed approximately 18% 1-year mortality in matched patients without telemonitoring.^16^ The OSICAT [Telemonitoring versus standard care in heart failure: a randomised multicentre trial] randomized trial reported approximately 35% HF rehospitalization at 1 year;^17^ however, these findings are not directly comparable with our cohort because all patients had been hospitalized for HF within the preceding 12 months, representing a higher-risk population. As opposed to remote monitoring, which mostly focuses on detecting HF decompensation events, RM is a comprehensive approach that encompasses prevention, detection, and care. This difference underlies the reported reductions in HF hospitalization with RM.^5^^,^^18^ RM requires a multiparametric approach and necessarily involves a dedicated telemedicine staff, comprising nurses and cardiologists, in charge of evaluating patients’ statuses and the effect of outpatient interventions on a daily basis. For instance, blood pressure monitoring appears to be an essential component of RM strategies and has been shown to be associated with fewer HF rehospitalization, with strategies that did not measure blood pressure failing to demonstrate significant benefits.^19^ Specialized RM teams are tasked with ongoing patient education, the initiation and goal-directed titration of HF medical therapies, and prompt diuretic dose adjustments or other escalations in therapy in response to worsening HF events. This degree of organization has shown to be associated with fewer hospitalizations and mortality among HF patients, contrasting with simpler approaches in which alert management was delegated to less specialized health care providers and which failed to demonstrate benefit.^20^ Notably, the low rate of early study dropout reflects the robustness of this structured care pathway. Whereas previous study had a higher rate of patients living in MUAs being excluded for dropping out of the study, we believe that the intensive support provided within this RM program helped mitigate potential disparities in adherence and follow-up. One recent meta-analyses by Scholte et al.^21^ have reported reductions in all-cause mortality and HF hospitalizations with RM of HF patients. Among the studies analyzed in this meta-analysis, the TIM-HF2 [Efficacy of telemedical interventional management in patients with heart failure (TIM-HF2): a randomised, controlled, parallel-group, unmasked trial] trial is the only randomized trial to have demonstrated an improvement in survival with a multiparametric RM program. However, this study by Koehler et al.^5^ involved a centralized 24/7 telemonitoring center which may not reflect real-world RM implementation in most settings. Using an RM strategy that is more suitable for a real-life approach, we observed an annual mortality rate of 8.8%, which is comparable to those observed in the TIM-HF2 RM groups (8% in the RM group vs 12% in the control group). Moreover, the OSICAT randomized clinical trial showed reductions in all-cause mortality among patients with NYHA functional class III/IV symptoms, those who were socially isolated, and when ≥70% adherence in body-weight measurements was achieved.^17^ Our data demonstrate that the benefits seen with RM in these clinical trials can be achieved in real-word settings with telemedical centers working during typical standard working hours, and including among patients with severe HF symptoms and in MUAs. Several aspects of the RM strategy likely contribute to these favorable outcomes, including enhanced early identification of HF exacerbations, streamlined communication between patients and health care providers, and facilitated adjustments of medical therapy in response to patient-specific alerts. The use of specialized HF teams involving cardiologists and nurses expectedly ensures targeted and expert HF management as well. Collectively, the above data support integrating tailored RM into broader HF management strategies, emphasizing the importance of early detection and timely interventions to prevent clinical deterioration. Recently, the HERMeS [Evaluation of mobile health technology combining telemonitoring and teleintervention versus usual care in vulnerable-phase heart failure management: a multicentre, randomised controlled trial] trial demonstrated that combining daily telemonitoring with structured teleintervention during the postdischarge vulnerable phase significantly reduced cardiovascular events compared with usual care.^22^ This benefit was largely attributed to the ability of mHealth platforms to trigger timely, often unplanned contacts, allowing early therapeutic adjustments. In line with this result, our real-world findings showed that a similar RM program can achieve comparable effectiveness regardless of whether patients live in MUAs.

### Remote management of HF to improve health equity

Geographical accessibility to care providers represents an important barrier to health equity. In France, the estimated proportion of the overall population living in MUAs dramatically increased from 4% in 2005% to 18% in 2017.^23^^,^^24^ In our study we showed that 32% of patients live in MUAs. Importantly, the LPA scores in our study population span a wide and representative range of the national distribution across French municipalities. Furthermore, multiple factors including an aging physician demographic, shifts in clinical practices, the costs of healthcare provision, and the patient disease burdens are anticipated to lead to further declines in healthcare accessibility in the future, potentially exacerbating health inequities. MUA are associated with higher rates of adverse health outcomes, such as preventable hospitalizations, emergency department visits, and death. This is particularly true for cardiovascular conditions, including HF.^25^ Johnston et al. showed that rural residence was associated with a 40% higher preventable hospitalization rate and a 23% higher mortality rate, compared to urban residence. They also demonstrated that access to specialists, notably cardiologists, accounted for 55% and 40% of the rural-urban difference in preventable hospitalizations and mortality.^26^ Moreover, there is a strong correlation between shortages of GPs and access to specialist care in MUAs. In a previous study, patients living in MUAs had fewer overall medical visits and saw fewer medical specialists and more GPs for their care than their urban counterparts, partly because of greater travel distance and time compared to those living in urban areas.^27^^,^^28^ RM programs have emerged as a promising strategy to address local resource shortages. However, although RM initiatives are remotely coordinated by specialized HF teams, successful implementation theoretically requires local health care networks to support patient care, potentially posing a challenge in MUAs. Most prior evaluations of telehealth in underserved settings originate from rural regions of the United States and have primarily relied on telephone-based support, whereas evidence on RM within European health systems remains scarce.^29^ This review by Azizi Z et al. demonstrated that digital health interventions have the potential to enhance self-care and knowledge of patients with HF living in underserved rural areas without impact on clinical outcomes. In our study, approximately one-third of patients enrolled in the HF RM program resided in a MUA. Our data suggest that these patients are broadly comparable to those living outside of MUAs, although there was evidence of less optimized drug therapy (ie, lower angiotensin-converting enzyme inhibitors/angiotensin II receptor antagonists/sacubitril-valsartan use). This study demonstrated that RM program can be delivered consistently in regions with heterogeneous access to local health care providers. Despite geographic barriers and lower access to local health care providers, we found that patients in MUAs derived similar outcomes from RM when compared to patients living outside MUAs. We found no association between MUAs and all-cause mortality or unplanned HF hospitalization, neither in univariable nor multivariable analyses. These data align with our previous analysis of remote HF management during the COVID-19 “lockdown” in France, which demonstrated that most patients with HF decompensation could be managed at home.^4^ As mentioned previously, the OSICAT study similarly suggested a benefit of RM among socially isolated patients with HF, with a significant 40% relative risk reduction in the primary end point, including unplanned hospitalizations, and a significant 38% relative risk reduction of first hospitalization for HF.^17^ Although this study focused on the social aspect of isolation, it underlined the potential disproportionate value of RM programs in high-risk populations due to barriers to health care access.

### Study limitations

Our study design does not allow the causal effect of RM to be assessed, as all patients were enrolled in the RM program, without randomization and control arm. These results need to be confirmed in future prospective, randomized controlled studies. In our study, medical interventions implemented as part of the RM program were tailored to individual patient characteristics and based on clinical judgment. No standardized protocol was established to guide interventions in response to individual alerts. However, this approach is consistent with clinical practice given the complexity and nuances of HF management. Although well-established and used in similar studies, the definition of MUA used in our analysis was developed specifically for France and may not be generalizable to other countries. However, it is comparable to definitions used in other countries, including the United States.^30^ The LPA scores are not currently available for cardiologists. However, previous studies demonstrated a correlation between shortages in GPs and limited access to specialist care, suggesting that MUAs are also likely to encounter shortages in specialist availability.^27^^,^^28^ The timing and nature of patients alerts, and the management instituted in response to them were not recorded in available patient follow-up records. Therefore, data exploring the relationship between alerts, interventions, and clinical outcomes are unavailable for analysis. Although the adjudication process was rigorous and involved both prospective annotation within the RM platform and systematic review of all electronic health records, the possibility of missing a small number of events, particularly those occurring outside the regional hospital network, cannot be fully excluded. Despite the bicentric design of the study and the use of a standardized telemonitoring protocol across both centers, unmeasured intercenter heterogeneity cannot be completely ruled out.

## Conclusions

In a large cohort of HF patients enrolled in a structured, multiparametric RM program, this study demonstrated that RM program can be delivered consistently in regions with heterogeneous access to local healthcare providers. We showed a low rate of unplanned HF hospitalizations and all-cause mortality irrespective of MUA designation. Randomized, prospective, multicenter, controlled studies are needed to confirm these results.

Perspectives **COMPETENCY IN MEDICAL KNOWLEDGE:** This study demonstrates that a structured, multiparametric RM program for HF achieves similar clinical outcomes—specifically rates of unplanned HF hospitalization and all-cause mortality—regardless of whether patients reside in MUAs or not. These findings underscore the value of RM strategies in improving access to quality HF care. Clinicians should consider RM as an effective tool to provide equitable care across diverse practice settings.

**TRANSLATIONAL OUTLOOK:** Future research should focus on identifying the optimal components of RM programs, assessing cost-effectiveness in MUAs, and developing scalable models for broader deployment. Randomized trials are warranted to confirm these observational findings and guide national health policies.

## Funding support and author disclosures

This study received financial support from the French Government as part of the “Investments of the Future” program managed by the French National Research Agency (ANR), Grant reference ANR-10-IAHU-04. Drs Ploux and Eschalier are shareholders of Careline Solutions. All other authors have reported that they have no relationships relevant to the contents of this paper to disclose.

## Supplementary material

Supplementary docx 1
