# Supplementary material for: Remote Management of Patients With Heart Failure in Medically Underserved Areas
Source: JACC Adv. 2026 Mar 19;5(4):102676. doi: 10.1016/j.jacadv.2026.102676 (PMC13019588; doi:10.1016/j.jacadv.2026.102676)
Supplement: Supplementary docx 1 [file mmc1.docx]

**Remote Management of Heart Failure Patients in Medically Underserved Areas**

**Supplemental Materials**

**Table of Contents:**

- Supplemental Table 1. Missing data summary
- Supplemental Table 2: Diagnosis of the imputation procedure
- Supplemental Table 3: Remote monitoring alerts at the end of the follow-up in the overall population (N=1,040).
- Supplemental Table 4: Univariable analysis of clinical and remote monitoring characteristics to predict the primary outcome in the overall population (N=1,040).
- Supplementary Table 5. Complete-case Cox proportional hazards model (N=837)
- Supplemental Figure 1: Distribution of heart failure aetiologies other than ischaemic and dilated cardiomyopathies in the overall population (N=1,040).
- Supplemental Figure 2: Competing risk analysis for heart failure hospitalisation and all-cause mortality in the overall population (N=1,040).

# **Supplemental Table 1. Missing data summary**

| Variable | N missing data | % missing data |
| --- | --- | --- |
| Albumin | 171 | 16.4 |
| Haemoglobin | 170 | 16.3 |
| NTproBNP | 144 | 13.8 |
| Diabetes | 105 | 10.1 |
| Hypertension | 95 | 9.1 |
| Atrial fibrillation | 52 | 5.0 |
| Aldosterone antagonists | 51 | 4.9 |
| Furosemide | 36 | 3.5 |
| CIED | 32 | 3.1 |
| ACEi/ARB | 20 | 1.9 |
| eGFR | 17 | 1.6 |
| Betablockers | 12 | 1.2 |
| NYHA status | 2 | 0.2 |
| SGLT2i | 1 | 0.1 |

Caption: Missing data proportions for each variable are reported. Multiple imputation was performed using a random forest–based algorithm implemented in the “*missForest”* package. Ten imputed datasets were generated. For each imputation, missing values were iteratively updated using random forest models trained on observed data. The maximum number of iterations was set to 10 (maxiter = 10). Convergence was assessed internally by monitoring the normalised root mean squared error (NRMSE) between successive iterations, and stabilisation was observed before reaching the maximum number of iterations. Each forest consisted of 200 trees (ntree = 200) with bootstrap sampling (replace = TRUE). The number of candidate variables randomly selected at each split (mtry) and node size were kept at their default values, as recommended in the original methodological description of the algorithm. Cox proportional hazards models were fitted within each imputed dataset, and parameter estimates were pooled according to Rubin’s rules.

Abbreviations: ACEi, angiotensin-converting enzyme inhibitor; ARB: angiotensin II receptor CIED, cardiac implantable electronic device; eGFR, estimated glomerular filtration rate; NT-proBNP, N-terminal pro–B-type natriuretic peptide; NYHA, New York Heart Association; SGLT2i, sodium–glucose cotransporter 2 inhibitor.

# **Supplemental Table 2. Diagnosis of the imputation procedure**

| Variable | Type | NRMSE | PFC |
| --- | --- | --- | --- |
| CEID | Categorical |  | 0.245 |
| ACEi/ARB | Categorical |  | 0.205 |
| Betablockers | Categorical |  | 0.197 |
| Aldosterone antagonists | Categorical |  | 0.117 |
| NYHA status | Categorical |  | 0.181 |
| Diabetes | Categorical |  | 0.151 |
| Hypertension | Categorical |  | 0.114 |
| Atrial fibrillation | Categorical |  | 0.187 |
| SGLT2i | Categorical |  | 0.159 |
| Albumin | Numeric | 0.198 |  |
| Haemoglobin | Numeric | 0.196 |  |
| NTproBNP | Numeric | 0.195 |  |
| Furosemide | Numeric | 0.192 |  |
| eGFR | Numeric | 0.117 |  |

Caption: Imputation accuracy was assessed using out-of-bag error estimates derived from the random forest algorithm. For continuous variables, imputation error is reported as the NRMSE, calculated as the square root of the mean squared error divided by the standard deviation of the observed values. For categorical variables, imputation error is reported as the PFC values. Lower values indicate better imputation performance.

Abbreviations: ACEi, angiotensin-converting enzyme inhibitor; ARB, angiotensin II receptor blocker; CIED, cardiac implantable electronic device; eGFR, estimated glomerular filtration rate; NRMSE, normalised root mean squared error; NT-proBNP, N-terminal pro–B-type natriuretic peptide; NYHA, New York Heart Association; PFC, proportion of falsely classified; SGLT2i, sodium–glucose cotransporter 2 inhibitor.

**Supplemental Table 3: Remote monitoring alerts at the end of the follow-up in the overall population (N=1,040).**

|  | **All patients** | **No MUA** | **MUA** | **p-value** |
| --- | --- | --- | --- | --- |
|  | **(N=1,040)** | **(n=707)** | **(n=333)** |  |
| **Remote monitoring alerts, mean ± SD** |  |  |  |  |
| Total | 83.1 ± 86 | 80.1 ± 83.1 | 89.3 ± 91.6 | 0.121 |
| Body weight | 50 ± 66 | 48.4 ± 64 | 53.5 ± 70 | 0.262 |
| Blood pressure | 13.8 ± 11.6 | 14.3 ± 13.1 | 12.5 ± 7.28 | 0.776 |
| Heart rate | 19.2 ± 10.1 | 19.8 ± 9.5 | 17.9 ± 11.2 | 0.782 |
| Symptoms | 22.9 ± 29.6 | 21.1 ± 27.7 | 26.8 ± 33 | **0.006** |
|  |  |  |  |  |

Caption: Values are mean ± SD. Bold values indicate the 2-tailed p-value reached statistical significance (<0.05).

^a^ Remote monitoring alters corresponds to each parameter alert measured from the patient and transmitted to the medical team, used to manage patients.

Abbreviation: MUA, medically underserved areas; SD, standard deviation.

**Supplemental Table 4. Univariable analysis of clinical and remote monitoring characteristics to predict the primary outcome in the overall population (N=1,040).**

|  | **Hazard Ratio**  **(95% CI)** | **p-value** |
| --- | --- | --- |
|  |  |  |
| Age, years | 1.02 (1.01-1.03) | **0.002** |
| Male | 1.07 (0.77-1.47) | 0.689 |
| MUA | 0.97 (0.71-1.32) | 0.829 |
| NYHA >II | 1.64 (1.23-2.18) | **0.001** |
| LVEF, per 5% | 1.05 (0.99-1.11) | 0.055 |
|  |  |  |
| **HF aetiologies** |  |  |
| Others | Ref. |  |
| Dilated | 0.88 (0.58-1.33) | 0.538 |
| Ischaemic | 1.15 (0.83-1.58) | 0.403 |
|  |  |  |
| **Comorbidities** |  |  |
| Diabetes | 1.17 (0.88-1.56) | 0.282 |
| Hypertension | 1.1 (0.8-1.49) | 0.561 |
| Chronic kidney disease^a^ | 2.05 (1.48-2.84) | **<0.001** |
| Atrial fibrillation | 1.17 (0.85-1.6) | 0.336 |
|  |  |  |
| **Biology** |  |  |
| eGFR, per 10mL/min/1.73m2 | 0.83 (0.77-0.89) | **<0.001** |
| NT-proBNP, per 500pg/mL | 1.05 (1.04-1.06) | **<0.001** |
| Haemoglobin, per 1g/dL | 0.83 (0.77-0.9) | **<0.001** |
| Albumin, per 1g/L | 0.97 (0.96-0.98) | **<0.001** |
|  |  |  |
| **HF treatments** |  |  |
| ACEi/ARB/sacubitril-valsartan | 0.63 (0.45-0.87) | **0.005** |
| Beta-blockers | 0.77 (0.54-1.08) | 0.133 |
| Aldosterone antagonists | 0.74 (0.56-0.98) | **0.031** |
| SGLT2 inhibitors | 1.31 (0.85-2.03) | 0.216 |
| Digoxine | 0.85 (0.21-3.42) | 0.817 |
| Furosemide, per 40mg | 1.06 (1.03-1.08) | **<0.001** |
|  |  |  |
| **Cardiac devices** |  |  |
| Permanent pacemaker | 1.15 (0.71-1.86) | 0.567 |
| ICD | 0.69 (0.47-0.98) | **0.048** |
| CRT-P | 0.75 (0.35-1.63) | 0.468 |
| CRT-D | 0.62 (0.42-0.93) | **0.021** |
|  |  |  |
| **Remote monitoring parameters** |  |  |
| Blood pressure | 0.96 (0.36-2.6) | 0.942 |
| Heart rate | 0.7 (0.52-0.96) | **0.026** |
| Symptoms | 0.49 (0.29-0.84) | **0.009** |
| Cardiac devices | 0.88 (0.48-1.62) | 0.679 |
| Remote monitoring adherence, per 1% | 1.01 (0.99-1.01) | 0.61 |
|  |  |  |

Caption: Values are HR (95%CI). Bold values indicate the 2-tailed p-value reached statistical significance (<0.05).

^a^ Defined by eGFR ≤60ml/min/1.73m^2^ twice.

Abbreviations: ACEi: angiotensin-converting enzyme; ARB: angiotensin II receptor; BNP: B-type natriuretic peptide; CRT: cardiac resynchronisation therapy; eGFR: estimated glomerular filtration rate; ICD: implantable cardioverter defibrillator; HF: heart failure; LVEF: left ventricular ejection fraction; MUA, medically underserved area; NT-proBNP: N terminal pro brain natriuretic peptide; NYHA: New York Heart Association scale functional classification; SGLT: sodium–glucose cotransporter 2 inhibitors.

# **Supplemental Table 5. Complete-case Cox proportional hazards model (N=837)**

| Variable | HR | 95% CI | p-value |
| --- | --- | --- | --- |
| Age, years | 1.005 | 0.987–1.023 | 0.585 |
| MUA | 0.970 | 0.698–1.350 | 0.859 |
| Furosemide, per 40mg increase | 1.043 | 1.015–1.073 | **0.003** |
| Other aetiologies of HF^a^ | 0.718 | 0.482–1.070 | 0.104 |
| Atrial fibrillation | 1.096 | 0.752–1.598 | 0.633 |
| NYHA status >II | 1.273 | 0.922–1.758 | 0.142 |
| LVEF, per 5% increase | 1.009 | 0.996–1.023 | 0.168 |
| Diabetes | 1.257 | 0.899–1.759 | 0.181 |
| Hypertension | 0.881 | 0.615–1.263 | 0.491 |
| eGFR, per 10mL/min/1.73m^2^ increase | 0.944 | 0.867–1.028 | 0.182 |
| NTproBNP, per 500pg/mL increase | 1.039 | 1.024–1.055 | **<0.001** |

Caption: HRs with 95%CIs derived from a Cox proportional hazards model including only individuals with complete data on all covariates. Bold values indicate the 2-tailed p-value reached statistical significance (<0.05).

^a^ Other aetiologies of HF correspond to non-ischemic nor dilated aetiologies of HF.

Abbreviations: Same as Supplemental Table 4.

**Supplemental Figure 1. Distribution of heart failure aetiologies other than ischemic and dilated in the overall population (N=1,040).**

**
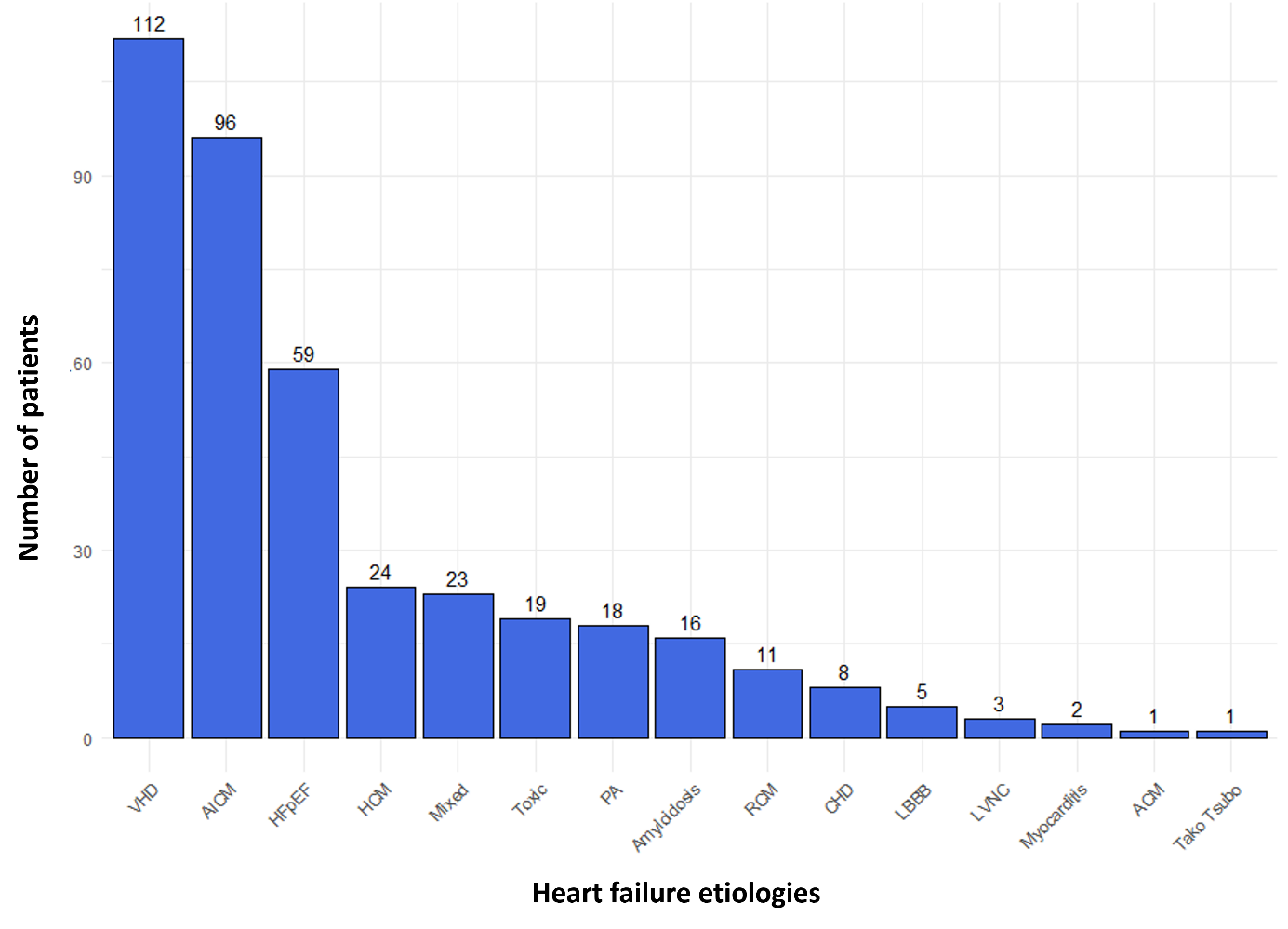
**

Caption: Distribution of the number of patients for each heart failure aetiologies (without ischemic and dilated cardiomyopathies). Mixed aetiology is defined by at least 2 different aetiologies (for example, valvular heart disease and pulmonary hypertension).

Abbreviation: ACM, arrhythmogenic cardiomyopathy; AICM, arrythmia-induced cardiomyopathy; CHD, congenital heart disease; HCM, hypertrophic cardiomyopathy; HFpEF, heart failure with preserved ejection fraction; LBBB, left bundle branch block; LVNC, left ventricular non compaction; PA, pulmonary arterial hypertension; RCM, restrictive cardiomyopathy; VHD, valvular heart disease.

**Supplemental Figure 2: Competing risk analysis for unplanned heart failure hospitalisation and all-cause mortality in the overall population (N=1,040).**


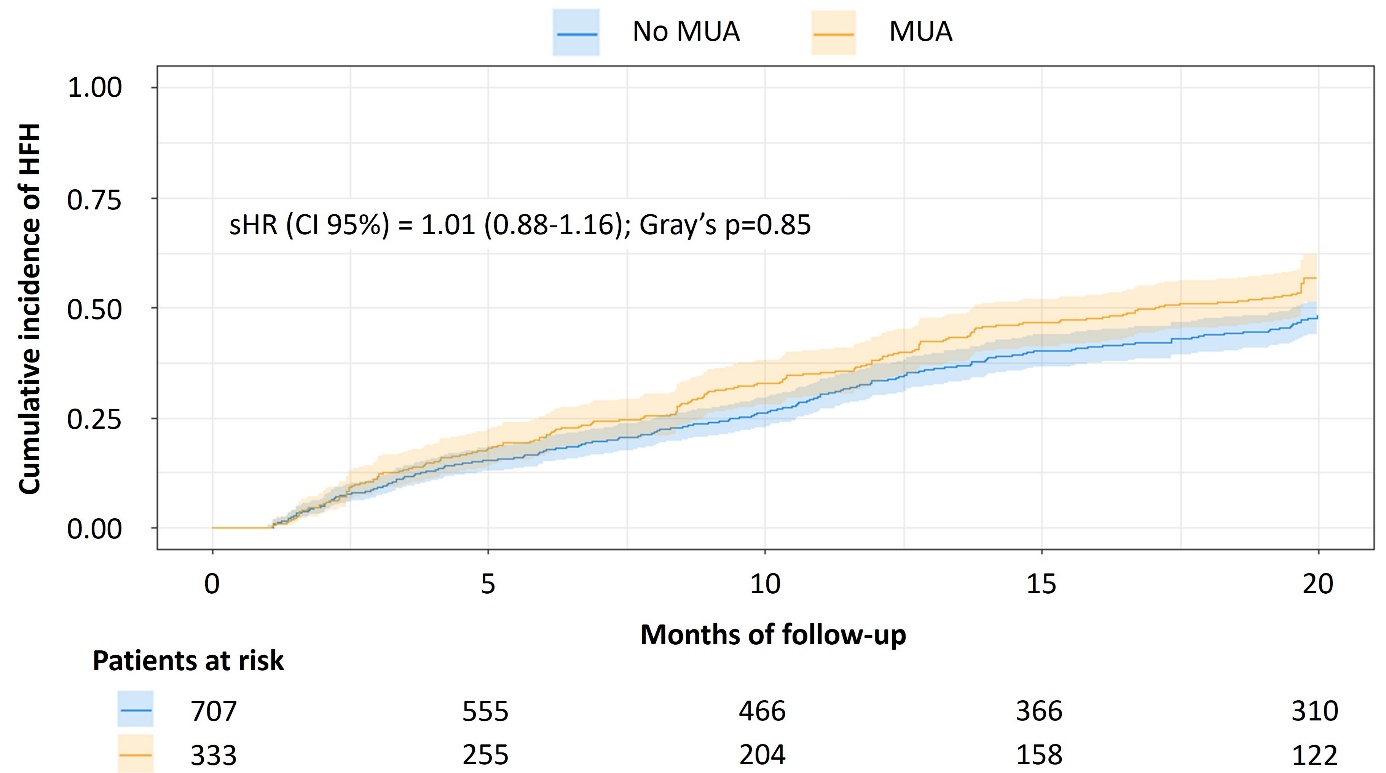


Caption: Cumulative incidence functions of heart failure hospitalisation with all-cause mortality treated as competing risk. Test comparing the test was based on the Gray’s test.

Abbreviations: HFH, heart failure hospitalisation; sHR: subdistribution hazard ratio.
